# Referral Processes and Dispositions in a Multidisciplinary Team for Older Adults With Cognitive Vulnerability

**DOI:** 10.1093/geroni/igab046.1262

**Published:** 2021-12-17

**Authors:** Richard Fortinsky, Kristen Annis-Brayne, Marie Smith, Kathleen Obuchon, Julie Robison, George Kuchel, Shawn Ladda

**Affiliations:** 1 University of Connecticut School of Medicine, Farmington, Connecticut, United States; 2 UConn Health, UConn Health, Connecticut, United States; 3 University of Connecticut, University of Connecticut, Connecticut, United States; 4 UConn Health, Farmington, Connecticut, United States; 5 University of Connecticut Health, Farmington, Connecticut, United States

## Abstract

Multidisciplinary team care for community-dwelling older adults with multiple chronic conditions has proven value. Older adults receiving team care experience better outcomes than by solo practitioners alone, and teams are being established as outgrowths of primary care and other clinical settings. Yet little is known about the inner workings of multidisciplinary teams, both in terms of how referral patterns among team members are established and the extent to which older adults and their families accept referrals from team leaders to other clinical disciplines within teams. In this presentation, we provide details about referral patterns and rates of acceptance by study participants in an ongoing clinical trial testing a multidisciplinary team designed to provide care management to older adults (age >65) with cognitive vulnerability due to dementia, depression, and/or delirium (3D Team). Nurse practitioners lead the 3D Team, conduct in-home clinical assessments and make referrals to other team members based on study protocols specifying participants’ eligibility for each 3D Team member. Results are based on the first 209 older adults randomized to the 3D Team. Pharmacist: all 209 members accepted having their medications reviewed and reconciled. Registered Dietician: of 134 referrals, 52 (38.8%) accepted. Occupational Therapist, of 117 referrals, 65 (55.6%) accepted. Physical Therapist: of 109 referrals, 92 (84.4%) accepted. Community Health Educator: of 106 referrals, 101 (95%) accepted. LCSW for depression-related problem solving therapy: of 76 referrals, 55 (72.4%) accepted. Criteria for referrals and interpretations of variations in referral acceptance rates by older adults and their families will be discussed.

